# Extracorporeal immune cell therapy of sepsis: ex vivo results

**DOI:** 10.1186/s40635-022-00453-8

**Published:** 2022-06-16

**Authors:** Gerd Klinkmann, Thomas Wild, Benjamin Heskamp, Fanny Doss, Sandra Doss, Lubomir Arseniev, Krasimira Aleksandrova, Martin Sauer, Daniel A. Reuter, Steffen Mitzner, Jens Altrichter

**Affiliations:** 1 Department of Anaesthesiology and Intensive Care Medicine, University of Rostock, Schillingallee 35, 18055 Rostock, Germany; 2 ARTCLINE GmbH, Schillingallee 68, 18057 Rostock, Germany; 3 Division of Nephrology, Department of Medicine, Medical Faculty, University of Rostock, Ernst-Heydemann-Str. 6, 18057 Rostock, Germany; 4 Department of Extracorporeal Therapy Systems, Fraunhofer Institute for Cell Therapy and Immunology, Schillingallee 68, 18057 Rostock, Germany; 5 Cellular Therapy Centre (CTC), Medizinische Hochschule Hannover, Feodor-Lynen-Str. 21, 30625 Hannover, Germany; 6 Center for Anesthesiology and Intensive Care Medicine, Hospital of Magdeburg, Birkenallee 34, 39130 Magdeburg, Germany; 7 Department of Anesthesiology and Intensive Care Medicine, University Medical Center Rostock, Schillingallee 35, 18057 Rostock, Germany

**Keywords:** Extracorporeal, Therapy, Sepsis, Clinical Use, Granulocyte, Concentrate

## Abstract

**Background:**

Immune cell dysfunction plays a central role in sepsis-associated immune paralysis. The transfusion of healthy donor immune cells, i.e., granulocyte concentrates (GC) potentially induces tissue damage via local effects of neutrophils. Initial clinical trials using standard donor GC in a strictly extracorporeal bioreactor system for treatment of septic shock patients already provided evidence for beneficial effects with fewer side effects, by separating patient and donor immune cells using plasma filters. In this ex vivo study, we demonstrate the functional characteristics of a simplified extracorporeal therapy system using purified granulocyte preparations.

**Methods:**

Purified GC were used in an immune cell perfusion model prefilled with human donor plasma simulating a 6-h treatment. The extracorporeal circuit consisted of a blood circuit and a plasma circuit with 3 plasma filters (PF). PF1 is separating the plasma from the patient’s blood. Plasma is then perfused through PF2 containing donor immune cells and used in a dead-end mode. The filtrated plasma is finally retransfused to the blood circuit. PF3 is included in the plasma backflow as a redundant safety measure. The donor immune cells are retained in the extracorporeal system and discarded after treatment. Phagocytosis activity, oxidative burst and cell viability as well as cytokine release and metabolic parameters of purified GCs were assessed.

**Results:**

Cells were viable throughout the study period and exhibited well-preserved functionality and efficient metabolic activity. Course of lactate dehydrogenase and free hemoglobin concentration yielded no indication of cell impairment. The capability of the cells to secret various cytokines was preserved. Of particular interest is equivalence in performance of the cells on day 1 and day 3, demonstrating the sustained shelf life and performance of the immune cells in the purified GCs.

**Conclusion:**

Results demonstrate the suitability of a simplified extracorporeal system. Furthermore, granulocytes remain viable and highly active during a 6-h treatment even after storage for 3 days supporting the treatment of septic patients with this system in advanced clinical trials.

## Introduction

### Background

Sepsis is defined as life-threatening organ dysfunction arising from a dysregulated host response to infection [1].

Immune dysfunction is a hallmark of sepsis and septic shock. This so-called "sepsis-induced immunoparalysis" is characterized by a phase of immunosuppression preceded or even paralleled by the early hyperinflammatory state [2]. Sepsis-induced immunoparalysis renders patients unable to clear their primary infection and exposes them to a higher risk of dying at a later stage from secondary or opportunistic infections in an immunosuppressed state [3–6]. Although its importance is increasingly recognized, there is still an absence of consensus on the relevance of this clinically emerging phenomenon [7, 8].

Despite our increasing understanding of the molecular and pathophysiologic processes underlying sepsis-related organ injury, all treatment options remain nonspecific with respect to host response [1]. In recent years, immunomodulation has been introduced as a complementary therapeutic strategy to overcome immune system dysfunction in sepsis [9]. The transfusion of granulocyte preparations failed to improve survival in sepsis and neutropenic patients [10, 11]. There is some indication that steroid- or G-CSF-stimulated high-yield granulocyte donations might result in better survival in severe infections associated with neutropenia and cancer. However, reported clinical benefits remain ambiguous [11, 12]

Similarly, a compelling biological rationale has emerged supporting the application of extracorporeal therapies based on our pathophysiologic understanding of sepsis. Extracorporeal therapies have been suggested to influence successfully immune imbalances and subsequently the clinical course of multiorgan failure and sepsis [13]. Some studies showed hemodynamic stabilization of patients during extracorporeal treatment of sepsis. However, no clear impact on survival was seen [14, 15]

Extracorporeal cell-based treatment is another promising approach emerging in this area. As such, cell perfusion therapies have been investigated for the treatment of liver failure and acute renal failure associated with sepsis using hepatocytes or renal tubule cells. Appropriate cell source selection appeared to be crucial in this regard. With regard to cellular immunocompetence, functional impairment of neutrophils and monocytes has been associated with increased mortality in advanced stages of sepsis [16–18]. Accordingly, the concept of using immune cells to provide extracorporeal treatment for sepsis-induced immunoparalysis arose from previous suggestions [19].

In order to deploy the beneficial features of neutrophils such as phagocytosis of cellular debris, antigenic material, or pathogens and at the same time to circumvent the possible damaging local effects of systemically transfused neutrophils, a bed-side cell perfusion therapy which uses immune cells (e.g., from granulocyte concentrates from healthy blood donors) in a strictly extracorporeal mode seems to be a promising therapeutic approach. The immune cells are retained in the extracorporeal system and discarded after the treatment, thus avoiding potential unwanted effects of granulocyte transfusions resulting from direct tissue contacts in the patient and his or her intravascular system and especially the endothelium.

The general rationale for such an approach is that on one hand the plasma-modifying capacity of human immune cells can be used (e.g., to remove antigenic material from the circulation, immune regulation via adsorption and secretion of cytokines) while on the other hand control over these cells can be maintained (e.g., retention of the cells in the extracorporeal circuit, preventing local tissue effects).

Granulocyte concentrates have already been investigated as cell perfusion therapy both preclinically and in pilot clinical trials, demonstrating safety and tolerability [20–22]. Recently, we reported a novel method to yield a high-purity, neutrophil-rich, functional leukocyte preparation from standard granulocyte concentrates. By developing this procedure, storage of GC was extended from 1 day to at least 3 days with preserved granulocyte viability and function. Furthermore, combination of longer storage time, lower cell count variation and ABO independence has the potential to offer purified GC as off-the-shelf immune cell preparations [23]. Thus, for the first time, a pure leukocyte population is recruitable for intensive care settings.

It is the aim of this ex vivo study to demonstrate the suitability of these purified GCs in a simplified extracorporeal treatment system in preparation for clinical trials in patients suffering from sepsis and septic shock.

## Methods

### Donors

Standard GCs were obtained from volunteer healthy donors from the donor pools of the Institute of Transfusion Medicine and Transplant Engineering (Hannover Medical School, Germany) according to national and local GMP guidelines for donation of granulocyte concentrates. All donors met the screening and testing criteria for granulocytapheresis defined by the Guidelines for the preparation of blood and blood components and the use of blood products of the German Medical Association [24]. Following informed consent, donors were stimulated as follows by a single subcutaneous injection of G-CSF 6 µg/kg body weight (Lenograstim, Kohlpharma, Merzig, Germany) and 8 mg oral dexamethasone approximately 16–18 h before granulocytapheresis.

### Granulocyte apheresis, purification

Standard GC were collected by continuous-flow apheresis with the COBE Spectra OPTIA™ Apheresis System (Terumo BCT Europe, Garching, Germany). Samples collected from the standard GC were tested for blood cell counts, viability and functionality.

**Fig. 1:**
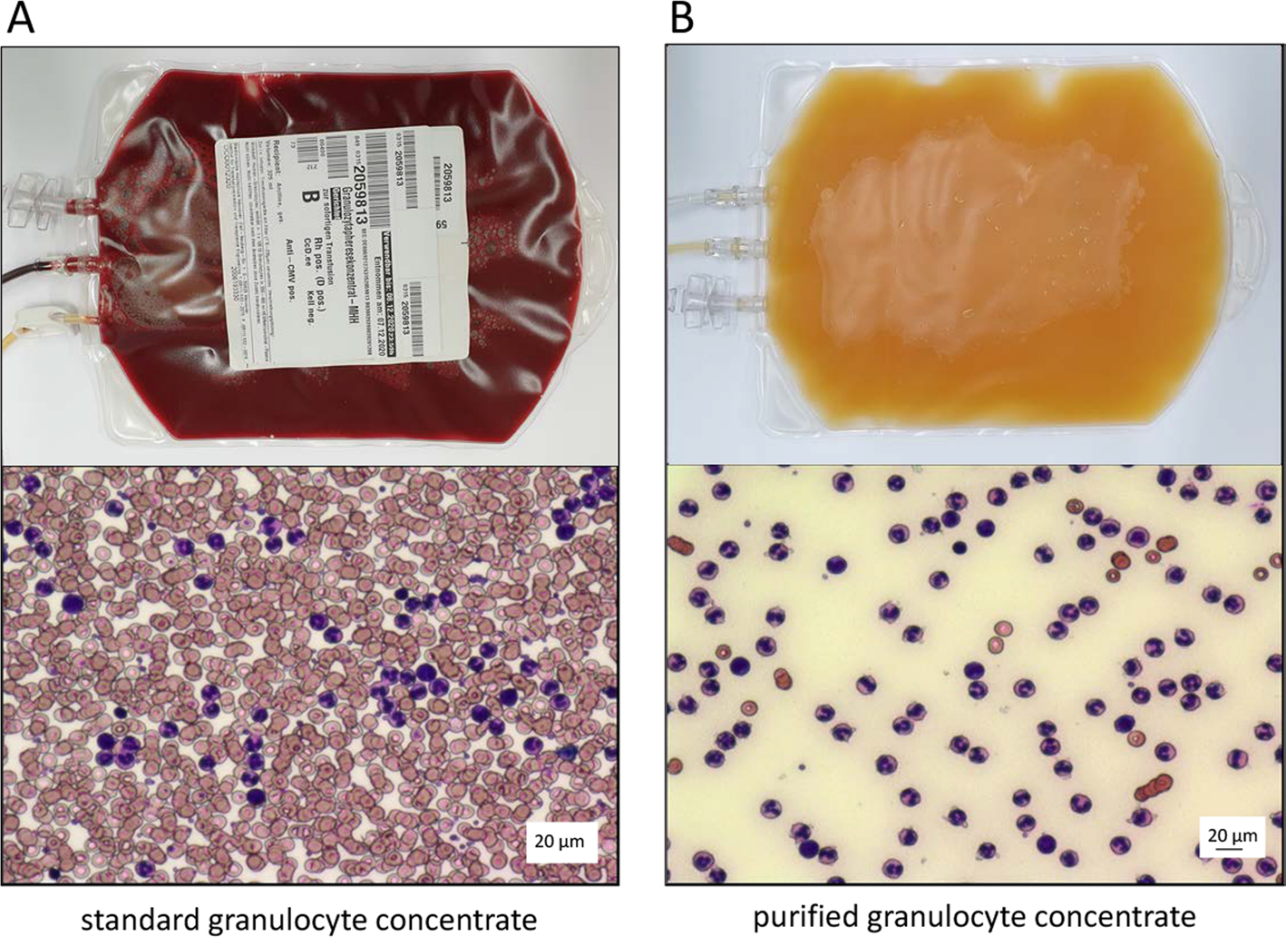
Appearance and histology (×400) of **A** standard GC and **B** purified GC. The higher purity and greatly diminished RBC count in the purified GC is evident from its yellow color resulting from the few remaining RBC and platelets

Development of the purification process and the successful extension of storage time to at least 3 days have been described recently. In short, standard GC were sedimented to remove red blood cells, washed to remove platelets and stored in ABO-compatible, citrate anticoagulated blood plasma with glucose in gas permeable storage bag. Each resulting purified GC unit contains at least 1 × 10^10^ granulocytes purified GC were stored at room temperature (22 ± 2 °C) without agitation [23]. Purification was performed according to national and local GMP guidelines at Cellular Therapy Centre (Hannover Medical School, Germany). Figure 1 provides a photograph of both the standard GC and the purified GC.

### In vitro plasma perfusion

The intended use of the purified GC is the therapeutic application in an extracorporeal treatment system to treat patients in septic shock. In essence, the laboratory model of extracorporeal immune cell therapy was similar to the clinical application, except for the presence of a genuine subject. A standardized 1000 mL plasma pool from donors was used as a model subject. Frozen plasma was thawed in a water bath at 37 °C until it was connected to the system.

**Fig. 2:**
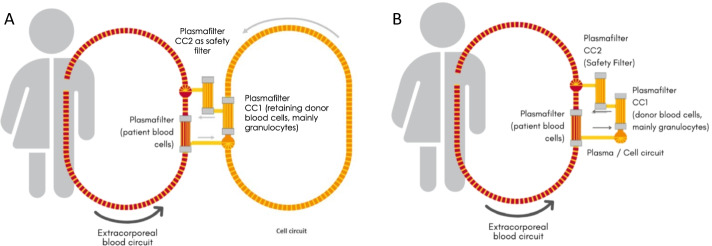
The extracorporeal immune cell therapy is a plasma treatment technology. Plasma is continuously filtered from the patient´s extracorporeal blood circuit and transferred into a closed-loop ‘cell circuit’, where the patient’s plasma is brought into direct contact with therapeutically effective, human-donor immune cells (i.e. the granulocyte concentrate). **A** Illustrates the procedure of separating plasma from an extracorporeal blood circuit through a plasma filter and feeding the plasma into a second circuit with circulating granulocytes as it has been used in clinical trials using standard granulocyte concentrates **B** illustrates the streamlined “one way” immune cell perfusion method using purified granulocyte concentrates. Plasma filter CC2 serves as a redundant safety filter

The CE marked disposable tubing set (Meise GmbH, Schalksmühle, Germany) and plasma filters (Medica S.p.A., Medolla, Italy) were connected to form a closed, sterile system. This system was filled and rinsed with a hemofiltration fluid (MultiBic 4 mmol/L Kalium, Fresenius Medical Care, Bad Homburg, Germany). Figure 2 provides a schematic illustration of the therapeutic setup.

Hemofiltration solution used for rinsing had following contents:

**Table:** 

K^+^: 4.0 mmol/L	Cl^−^: 111 mmol/L
Na^+^: 140 mmol/L	HCO_3_^−^: 35 mmol/L
Ca^2+^: 1.5 mmol/L	Glucose: 5.55 mmol/L
Mg^2+^: 0.5 mmol/L	

**Fig. 3:**
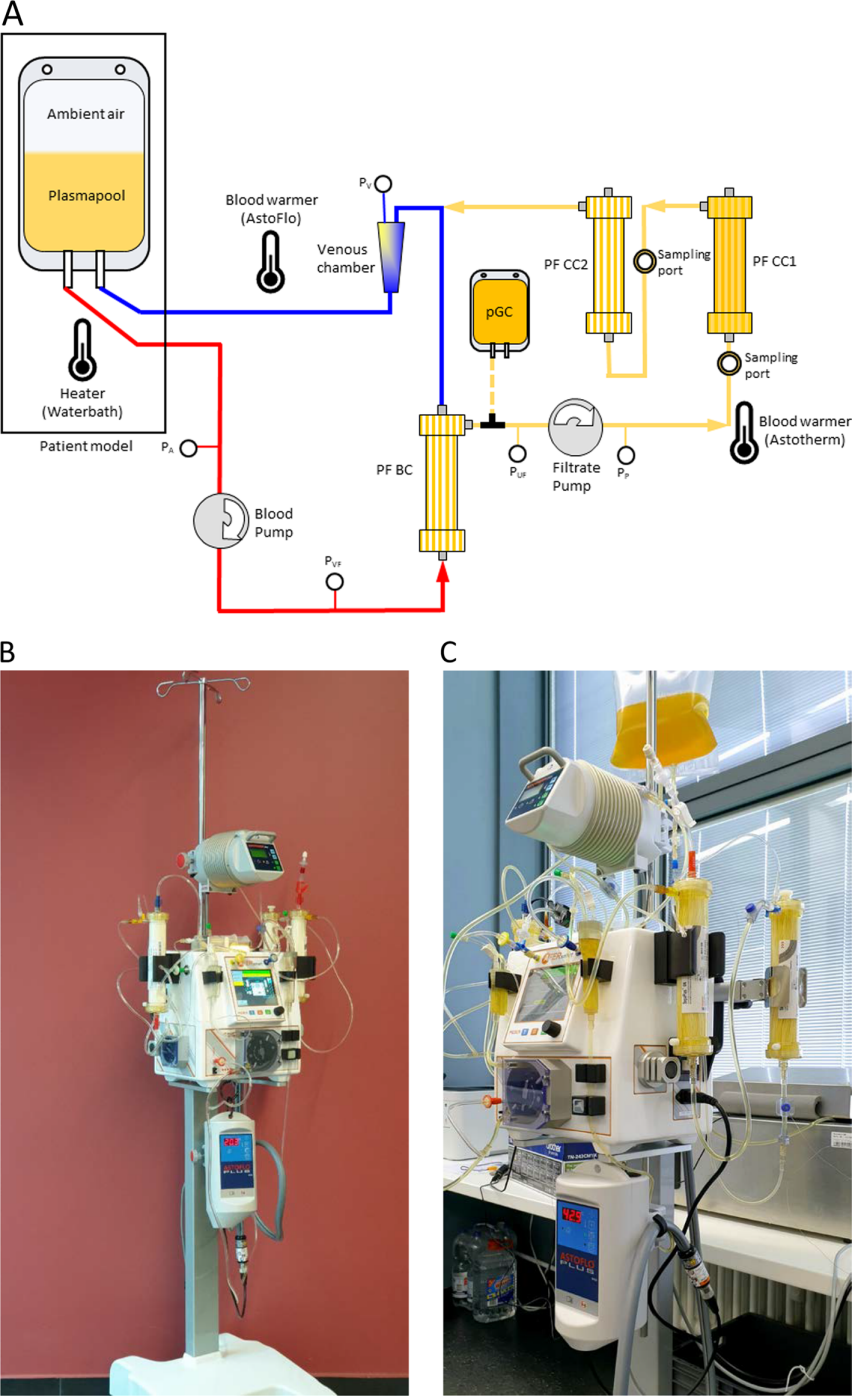
**A** Schematic of the treatment simulation experiments depicts a graphical representation of the components involved in the immune cell enhancement single-pass therapy system. In vitro treatment simulation.** B** Apheresis machine AFERsmart with blood warmers and installed tubing set.** C** Simulation experiment using a purified GC preparation

Heparin was added as anticoagulant in a concentration of 5 IU/mL. After rinsing the assembled tubing and filter system, purified GC preparation was heparinized with 10 IU/mL and subsequently filled into the plasma part of the tubing system. The rinsing solution was displaced by the volume of the cell preparation (approx. 400–450 mL). The cells remained inside the hollow fibers of a plasma filter (PF CC1) during the treatment. The system was then connected to a plasma pool, which was adjusted to 20 IU/mL heparin and 1.6–2.0 mmol/L free Ca^2+^ ions. The intended concentration of free Ca^2+^ ions in the extracorporeal circulation was > 1.0 mmol/L. Since purified GC cell preparations contain sodium citrate as anticoagulant and therefore almost no free Ca^2+^ ions, the initial value in the plasma pool was set correspondingly higher to reach a level of > 1.0 mmol/L after mixing. Glucose concentration was set to 3.5–7.0 mmol/L. Total fluid volume including the plasma pool was about 1850 mL. The different components mixed completely in the first 30 min of treatment simulation. In treatment mode, pool plasma enters the plasma filter PF BC (150 mL/min), where plasma is separated (33 mL/min). This plasma perfuses the plasma filter PF CC1 with the contained treatment cells inside in dead-end filtration mode. Subsequently, the plasma flows through another plasma filter PF CC2 in dead-end filtration mode, which acts as a safety barrier in case of a membrane rupture in PF CC1 to avoid donor cells entering the patient. After this, treated plasma flows into the venous chamber where it is mixed with the “patient´s blood” and returned to the “patient”. This treatment is performed continuously for 6 h. Samples from plasma stream were taken before and after cell filter PF CC1 after every hour. After 3 and 6 h the cell filter was flushed back to obtain cells for analysis. 10 extracorporeal experiments were performed and analyzed. A schematic illustration of the circuit is presented in Fig. 3A.

### Sampling

Samples were collected at different sample ports in the circuits and time points. Sampling time points were: before start, after 10 min, 30 min, 1 h, 2 h, 3 h, 4 h, 5 h, 6 h. Sampling ports were: before and after the cell filter PF CC1. To obtain cells sticking in the plasma filter PF CC1 after 3 h a sample was taken by backwashing plasma filter PF CC1 with 50 mL plasma and the 6 h sample by backwashing with 2000 mL cold (4 °C) NaCl solution at end of circulation.

Samples were taken and analyzed to determine leukocyte recovery rate, viability and functional capacity. Furthermore pH, electrolyte, glucose, lactate, lactate dehydrogenase and free hemoglobin concentrations as well as oxygen and carbon dioxide partial pressure were determined.

At each filter, the pressure before and behind the plasma filter membrane was monitored over the whole treatment duration of 6 h. For verification of donor cell retention, the fluid behind the membrane of plasma filter PF CC1 was collected, centrifuged and concentrated and then examined. Figure 3B and C provides photographs of the system.

### Measurement of granulocyte function

Function of the granulocytes was analyzed with oxidative burst and phagocytosis assays using commercial Phagoburst-Kit and Phagotest-Kit (Celonic, Heidelberg, Germany), respectively. Both tests were used according to manufacturer’s instructions with one modification, because granulocyte concentration in GC is approximately 10 times higher than in whole blood. To achieve a concentration of approximately 5000 granulocytes/μL and therefore the same ratio of granulocytes to the stimulus (e.g., Escherichia coli) like with heparin-anticoagulated blood (4000–10,000 granulocytes/μL), samples were diluted in heparin-anticoagulated blood group compatible plasma of healthy donors.

### Measurement of cytokine concentration

Measurement of cytokine concentrations was performed by use of the LEGENDplex™ Human Essential Immune Response Panel (13-plex, BioLegend, Amsterdam, Netherlands). The panel is a bead-based multiplex assay panel that uses fluorescence-encoded beads suitable for use on various flow cytometers. Immunoassay was performed according to manufacturer's instructions. Quantitative analysis was carried out by a flow cytometer (MACS Quant 16, Miltenyi Biotec, Bergisch-Gladbach, Germany) and analysis was performed using LEGENDplex v8.0 software.

### Measurement of blood cell counts and WBC viability

WBC viability was determined using NucleoCounter® NC-200™ (ChemoMetec, Allerod, Denmark) according to manufacturer's specifications.

Blood cell content and white blood cell differentiation were evaluated automatically using a hematology analyzer (KX-21 N, Sysmex, Norderstedt, Germany).

Values at time point 3 h are measured in samples that were taken by drawing back a volume of 50 mL from PF CC1. By this method, about 4% of the original cell count were retrieved, mixed, a sample was taken, and the rest of the cells was re-introduced to PF CC1. The values at time point 6.5 h are measured in sample that were taken at the end of the experiment by flushing back PF CC1 with a volume of 2000 mL cold sodium chloride solution. By this method, an average of 46% of the original cell count were retrieved, mixed, a sample was taken and analyzed. Therefore, cell-associated results at 3 h and 6 h do not necessarily represent total cell amount.

### Measurement of electrolytes, pH, glucose, oxygen and carbon dioxide partial pressures

Electrolytes, pH, glucose, oxygen and carbon dioxide partial pressures were measured using an ABL90 Flex Plus blood gas analyzers (Radiometer, Krefeld, Germany).

### Measurement of lactate, and lactate dehydrogenase and free hemoglobin concentrations

Concentrations of lactate, free hemoglobin (fHb) and lactate dehydrogenase (LDH) were determined using a Cobas Mira Plus CQ (Roche, Ludwigsburg, Germany) according to manufacturer's specifications. fHb concentration was measured using the 3-wavelength method (380/415/450 nm) according to the method of Harboe [25] on the spectral photometer Dr. Lange LS 500 (Type LPG 244) according to manufacturer’s specifications.

### Pressures

Transmembrane pressures (TMP) of plasma filters were calculated from respective pressure values at the inlet of the plasma filter and its outlet(s) as TMP = Pre-membrane mean pressure – post-membrane pressure. TMP of PF BC (plasma filter blood circuit) was measured and displayed by the apheresis machine AFERsmart (Medica, Medolla, Italy). TMPs at PF CC1 and PF CC2 were determined by additional pressure measurements in the tubing set.

### Statistical analysis

Statistical analysis was performed using IBM SPSS Statistics (version 27, Chicago, IL, USA). Results are expressed as median ± standard deviation (SD) and range. According to the distribution of data (using Shapiro–Wilk test), Mann–Whitney test was used for two independent samples for continuous variables. Kruskal–Wallis test was used to test differences between multiple independent samples with non-normal underlying population distribution, and appropriate post hoc tests were applied if necessary. Statistical differences were considered significant at *P* value < 0.05.

## Results

Standard GC used to produce purified GC were prepared according to clinical standard procedures by granulocytapheresis from healthy donors and met the quality criteria of the guidelines of the German Medical Association [24]. All 10 circuit experiments were run for 6 h without technical problems.

### Granulocyte function and viability

**Fig. 4:**
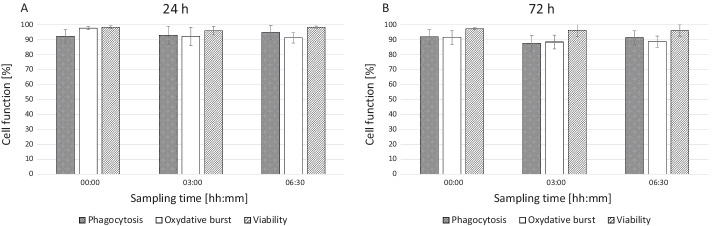
Cell function during the extracorporeal treatment simulation—phagocytosis, oxidative burst rate and viability (*n* = 10). The charts show the function parameters phagocytosis rate and oxidative burst rate of the WBC inside the treatment system before, during and after the simulated treatment. No significant differences were found between specific time points

Viability in purified GC was consistently high at over 94% across all subgroups during 6 h of treatment (Fig. 4A). Phagocytosis and oxidative burst assays demonstrated preservation of granulocyte function of purified GC in the circuit. Phagocytosis and oxidative burst remained preserved in the purified GC that were stored for 3 days prior to the circuit experiment (Fig. 4B).

### Glucose consumption and lactate generation

**Fig. 5:**
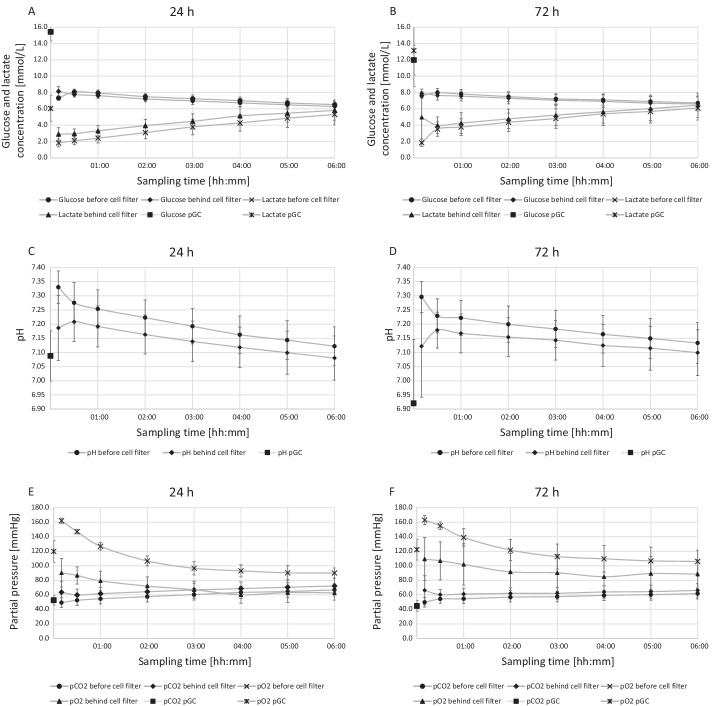
Glucose consumption and lactate generation during the extracorporeal treatment simulation (*n* = 10). Glucose is consumed continuously. Likewise, lactate is generated as a product of cell metabolism. Also, there is a difference in concentrations before and after cell filter CC1 until the end of the treatment, showing that the cells are still active. pH values pressures in the plasma before and after cell filter CC1 during the extracorporeal treatment simulation (*n* = 10). Purified GC have a low pH, especially on day 3. After mixing with the physiologically conditioned plasma pool, the resulting pH is about 7.2 and decreases continuously by the metabolic activity of the cells. Oxygen and carbon dioxide partial pressures in the plasma before and after cell filter CC1 during the extracorporeal treatment simulation (*n* = 10). Oxygen is consumed, and carbon dioxide is generated as a product of cell metabolism

During preparation of the purified GC glucose was supplemented to support a longer storage time. After storage for 1 day glucose concentration in purified GC amounts to 15.42 ± 1.13 mmol/L. After equilibration at *t* = 30 min within the circuit, a value of 8.06 ± 0.35 mmol/L before PF CC1 and 7.76 ± 0.31 mmol/L after PF CC1 was observed. Subsequently, the concentration decreased steadily to 6.52 ± 0.57 mmol/L before PF CC1 and 6.30 ± 0.60 mmol/L after PF CC1 at the end of the 6-h analysis interval. A comparable course was detected in the circuits with purified GC that were stored for 3 days prior to use. In particular, no significant differences were observed between these two cell populations (Fig. 5A, B).

After storage for 1 day, lactate concentration of the purified GC was 6.05 ± 1.62 mmol/L. At 30 min after initiation of the simulation, equilibrated values in the circuit are 2.10 ± 0.49 mmol/L before PF CC1 and 2.97 ± 0.58 mmol/L after PF CC1. At 6 h, after a continuous increase, levels have reached 5.31 ± 1.26 mmol/L before PF CC1 and 5.82 ± 1.21 mmol/L after PF CC1. However, no significant differences between the test sites before and after the cell filter were determined. A comparable course is seen after storage for 3 days (Fig. 5A, B).

### pH values

At baseline, mean pH of purified GC was 7.09 ± 0.09 after 1 day of storage, about 7.28 ± 0.07 after equilibration at *t* = 30 min and decreased to 7.12 ± 0.07 during 6 h of treatment. In comparison, after 3 days of storage pH in purified GC showed a mean value of 6.92 ± 0.23, about 7.23 ± 0.06 after equilibration at *t* = 30 min and decreased to 7.13 ± 0.07 during 6 h of treatment. Although a constant difference between the sample points before and after PF CC1 was detectable in all experiments with mean values of 0.06 after *t* = 30 min to 0.04 at *t* = 6 h no significance was reached (Fig. 5C, D).

### Oxygen and carbon dioxide partial pressures

Commencing at 162.00 ± 3.52 mmHg, the course of oxygen partial pressure before PF CC1 shows a decrease over the entire observation period to a value of 90.06 ± 7.00 mmHg after 6 h in the circuits after 1 day of storage. Oxygen partial pressures after PF CC1 exhibit considerably but non-significant lower values. Carbon dioxide partial pressures maintain a constant level during the entire circuit duration. After 3 days of storage, comparable courses of pO_2_ and pCO_2_ were detectable albeit at a slightly higher level (Fig. 5E, F).

### Impact on LDH and fHB dynamics

**Fig. 6:**
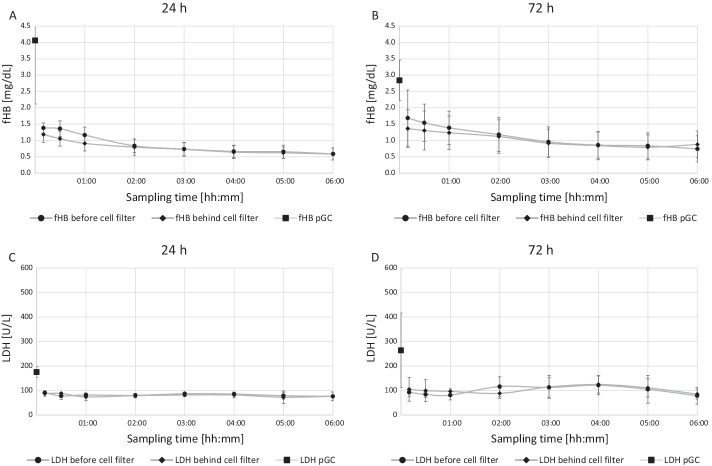
Free haemoglobin concentrations as an indication for erythrocyte damage (*n* = 10). Reference value is < 10 mg/dL. All values are very low and rather decreasing during the experiments. LDH activity as an indication for cell damage. Reference value is < 225 U/L (*n* = 10). All values are within the physiologic range and stable during the experiments

Figure 6A and B shows free hemoglobin concentrations as an indication for erythrocyte damage which in turn allows conclusions on viability of the granulocytes. In view of the reference value (< 10 mg/dL), all values were low and rather decreasing during the experiment. Similarly, Fig. 5C and D shows LDH concentrations as an indication for cell damage. Compared to the reference value (< 225 U/L), measured values were within the physiologic range and stable during the experiment

### Cytokine expression

**Fig. 7:**
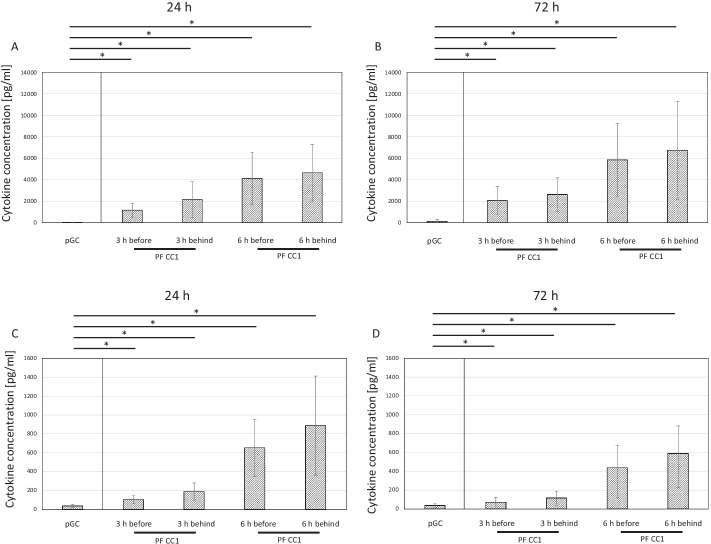
Interleukin-8 concentration pressures in the plasma before and after cell filter CC1 during the extracorporeal treatment simulation (*n* = 10). IL-8 level is low in the pGC and secreted during the extracorporeal treatment simulation both on day 1 and day 3. *P* values ≤ 0.05 (*) were considered significant. MCP-1 concentration pressures in the plasma before and after cell filter CC1 during the extracorporeal treatment simulation (*n* = 10). MCP-1 is low in the pGC and actively secreted during the extracorporeal treatment simulation both on day 1 and day 3. *P* values ≤ 0.05 (*) were considered significant

Interleukin-8 (IL-8) is a chemotactic factor that attracts neutrophils, basophils, and T-cells during the inflammatory process [26]. Monocyte chemoattractant protein-1 (MCP-1) is a potent monocyte-attracting chemokine [27]. IL-8 concentration of purified GC prior to initiation of the circuit simulation amounted to 14.78 ± 13.21 pg/mL (1 days of storage) and 115.39 ± 186.00 pg/mL (3 days of storage). During investigation, there was a significant increase up to values of 4648.67 ± 2637.27 pg/mL (1 day of storage) and 6744.53 ± 4547.30 pg/mL (3 days of storage) after 6 h (*P* < 0.05) (Fig. 7A, B). MCP-1 concentration of purified GC remained below 40 pg/mL for both cell populations before onset of the circuit simulation. During the experiment, there was a significant increase up to values of 887.63 ± 526.05 pg/mL (1 day of storage) and 587.01 ± 292.83 pg/mL (3 days of storage) after 6 h of treatment. Of particular interest is the fact that the cells performed the same on day 1 and day 3 supporting the extended storability of purified GC (Fig. 7C, D).

## Discussion

The main findings of this ex vivo investigation demonstrate that purified GC were for the first time successfully used in an extracorporeal immune cell plasma perfusion system. The preparations are thereby capable of being used at least until the end of the third day after donation and purification. Its potential therapeutic effect is sustained for a minimum of 6 h of extracorporeal plasma perfusion. Cytokine release dynamics may even argue for a longer treatment period.

While therapeutic plasma exchange has shown some positive signals in treatment of septic shock other extracorporeal blood purification techniques targeting the early hyperimmune “cytokine storm” phase have not yet been able to provide convincing evidence for routine clinical practice [13, 28, 29]. Indeed, this is reflected in the recommendation issued by the surviving sepsis campaign [1]. It is noteworthy that recently several clinical trials addressing early septic shock indicate a potential mortality risk for patients. For example, the COMPACT-2 trial, which investigated the use of high-volume coupled plasma filtration and adsorption in patients in septic shock, was terminated prematurely. Mortality was found to be 54% in the treatment group and 29% in the control group [30]. Likewise, hemoadsorption using the CytoSorb adsorber failed to provide positive results in patients in refractory septic shock [31]. Another clinical investigation examined COVID-19 patients suffering from severe acute respiratory distress syndrome who underwent extracorporeal membrane oxygenation. Additive cytokine adsorption increased mortality by 58% as a secondary outcome parameter [32]. A commonality of these trials is the therapeutic approach focusing on the early proinflammatory phase during sepsis. As an underlying principle of action, circulating mediators are removed from the patient's blood.

However, current intensive care supports septic patients through this early sepsis phase into a state referred to as immunoparalysis and characterized by, e.g., lymphopenia, low human leukocyte antigen DR (HLA-DR) on monocytes and impaired granulocyte function [9, 33]. Consequently, sepsis patients are at higher risk of mortality due to secondary infections and multiorgan failure. Therefore, a therapeutic principle that provides restorative support to the patient's dysregulated immune system represents one approach in solving this challenge [19, 34].

Different extracorporeal approaches have been proposed to address this issue and to successfully influence the immune system imbalance, thereby affecting the clinical course and outcome of multiorgan failure and sepsis. Specifically, David et al. reported the results of a randomized controlled trial evaluating additive therapeutic plasma exchange in patients with severe refractory septic shock and observed rapid hemodynamic improvement after this intervention confirming earlier results of Busund et al [28, 29]. Moreover, encouraging results have been obtained from pilot clinical trials evaluating the extracorporeal immune cell therapy as an additive cell-based approach in patients with severe refractory septic shock [20, 21]. There are two main concepts underlying this approach: on the one hand, the plasma-modifying ability of human immune cells is exploitable (e.g., removal of antigenic material from the circulation, immune regulation by adsorption and secretion of cytokines), and on the other hand, control over these cells can be maintained (e.g., retention of cells in the extracorporeal circulation, prevention of local tissue effects) [19]

Granulocyte concentrates were studied as extracorporeal cell perfusion therapy in a pilot phase I trial with ten septic shock patients and showed safety and compatibility of this complex therapy. The pilot trial was conducted as a prospective uncontrolled clinical phase I/II study with 28-day follow-up. The subjects were treated twice for 6 h within 3 days with about 1.5 × 10^10^ granulocytes from healthy donors. On average, about 10 L separated plasma were treated by the therapeutic donor cells. During treatments, bacterial endotoxin concentration showed significant reduction. Furthermore, noradrenaline dosage could be significantly reduced while mean arterial pressure was stable. Also, C-reactive protein, procalcitonin, and HLA-DR showed significant improvement. Four subjects died in hospital, six subjects could be discharged [20].

An extension of that trial including 9 subjects with septic shock and 1 subject with severe sepsis using about double the amount of granulocytes focused on the dosage of norepinephrine in subjects and influence on dynamic and cell-based liver tests during extracorporeal therapies. Extracorporeal treatment with donor granulocytes showed promising effects on dosage of norepinephrine in subjects, liver cell function, and viability in a cell- based biosensor [21]

A drawback of these trials was that a two-loop system requires substantial material input, complex apheresis equipment and personnel. Besides, such a process is demanding in handling. Further, immune cells are subjected to high stress due to constant movement of the cells through the tubes. Therefore, a streamlined “one-way” plasma perfusion system was developed compatible with rather simple apheresis equipment providing additional advantages like ease of use and less space requirement on ICU.

Foremost, the use of purified GC provides a significantly reproducible and narrower range of granulocytes per unit than obtained by standard GC. This enables precise therapy control in everyday clinical practice. Another aspect involves the performance of the cells in the circuit. A previous investigation comparing standard GC and purified GC in a recirculating system revealed WBCs of purified GC to settle in the filter after a short period of time, whereas many of the WBCs of standard GC moved continuously in the recirculating system throughout the entire treatment cycle [20]. This phenomenon appeared to be related to the interaction between WBCs and erythrocytes. These interactions potentially prevented adhesion of the WBCs in the filter. Considering the significantly lower amount of erythrocytes, these effects are minimized in the circuit of purified GC, which consequently leads to the persistence of cells in the filter. However, granulocytic activity including phagocytosis and oxidative burst were preserved completely. Consequently, continuous movement of cells in the circuit system is superfluous in the presence of purified GC. This could be confirmed in the present study using a streamlined "one-way" plasma perfusion system, which demonstrated high viability, phagocytosis, and oxidative burst throughout the entire treatment cycle. This observation is also consistent with IL-8 and MCP-1 concentration being low in baseline purified GC and increasing significantly during extracorporeal treatment simulation on both day 1 and day 3.

Lower erythrocyte and platelet counts in purified GC lead to lower lactate production and thus a more balanced pH in the circuit compared to standard GC in earlier studies. Use of special storage bags that release carbon dioxide produced by the cells during storage also contributes to better pH values of purified GC supplied to the circuit. Glucose is continuously consumed. Likewise, lactate is generated as a product of cell metabolism. Moreover, concentrations before and after the cell filter PF CC1 differ until the end of the treatment, indicating the cells remain active.

There are several limitations to the validity of these data. Since plasma filtration from whole blood is a standard technique and also for ethical reasons, the patient model was designed using human donor plasma rather than whole blood. Therefore, an ex vivo simulation of the treatment differs from clinical application in several parameters. While no patient was connected to the system, a plasma pool with a volume of 1000 mL was applied. Hence, the solute distribution volume was inferior to that in a clinical environment. A stationary mixture of water, electrolytes and blood proteins constituted the used plasma pool. In consequence, adsorption, resorption, and synthesis functions of a human body remained incompletely modeled. Additionally, in order to avoid technical problems with clotting in the rather static plasma pool bag, anticoagulation was performed with greater intent than in clinical applications. Oxygen supply to the cells was attenuated compared to a clinical situation with oxygen continuously supplied by the patient.

## Conclusion

The objective of the current study was to deploy purified GC in a streamlined extracorporeal plasma circuit designed for the treatment of septic patient. In conclusion, these results demonstrate that the streamlined "one-way" design entirely resembles previous results of the immune cell plasma perfusion system involving circulating immune cells with regard to immune cell functions including phagocytosis, oxidative burst, cytokine secretion, viability, and metabolism. Furthermore, fully preserved functionality of purified granulocyte concentrates stored for 3 days could be confirmed. Therefore, subsequent clinical trials with the extracorporeal immune cell therapy can be applied by the streamlined “one way” system with the purified granulocyte concentrates stored for at least up to 3 days prior to extracorporeal use. In 2022, a multicenter randomized controlled trial with the one-way system will be started in septic shock patients.

## Data Availability

The datasets used and analyzed during the current study are available from the corresponding author on reasonable request.

## Abbreviations

BC: Blood circuit
CC: Cell circuit
CE: Conformité européenne
GC: Granulocyte concentrates
G-CSF: Granulocyte-colony stimulating factor
GMP: Good Manufacturing Practice
NaCl: Sodium chloride
PF: Plasma filters
